# Multi-omics and single-cell analysis reveals machine learning-based pyrimidine metabolism-related signature in the prognosis of patients with lung adenocarcinoma

**DOI:** 10.7150/ijms.107694

**Published:** 2025-02-18

**Authors:** Tong Hu, Run Shi, Yangyue Xu, Tingting Xu, Yuan Fang, Yunru Gu, Zhaokai Zhou, Yongqian Shu

**Affiliations:** 1 Department of Oncology, The First Affiliated Hospital of Nanjing Medical University, Nanjing, China.; 2 Department of Urology, The First Affiliated Hospital of Zhengzhou University, Zhengzhou, China.; 3 Collaborative Innovation Center for Cancer Personalized Medicine, Nanjing Medical University, Nanjing, China.

**Keywords:** pyrimidine metabolism, machine learning, prognosis, multi-omics analysis, single-cell analysis, lung adenocarcinoma

## Abstract

**Background:** Pyrimidine metabolism is a hallmark of tumor metabolic reprogramming, while its significance in the prognostic and therapeutic implications of patients with lung adenocarcinoma (LUAD) still remains unclear.

**Methods:** In this study, an integrated framework of various machine learning and deep learning algorithms was used to develop the pyrimidine metabolism-related signature (PMRS). Its efficacy in genomic stability, chemotherapy and immunotherapy resistance was evaluated through comprehensive multi-omics analysis. The single-cell landscape of patients between PMRS subgroups was also elucidated. Subsequently, the biological functions of LYPD3, the most important coefficient factor in the PMRS model, were experimentally validated in LUAD cell lines.

**Results:** The PMRS model with “random survival forest” algorithm exhibited the best performance and was utilized for further analysis. It displayed excellent accuracy and stability in various model evaluation assays. Compared to the PMRS-high subgroup, patients with lower PMRS scores had better survival outcomes, more stable genomic characteristics and higher sensitivity to immunotherapy. Single-cell analysis indicated that as PMRS increased, epithelial cells gradually exhibited malignant phenotypes with enhanced pyrimidine metabolism, while PMRS-high patients showed an inhibitory status of tumor immune microenvironment. Further experiments indicated that LYPD3 promoted the malignant progression in LUAD cell lines.

**Conclusion:** Our study constructed the PMRS model, highlighting its potential value in the treatment and prognosis of LUAD patients and providing new insights into the individualized precision treatment for LUAD patients.

## Introduction

Lung cancer, the most frequently diagnosed cancer with the highest mortality rate globally, seriously threatens human health 1. Despite the recent advances in surgical resection, chemotherapy and immunotherapy, the 5-year survival rate for lung cancer still remains less than 20% 1,2. Lung adenocarcinoma (LUAD) is the most common histological subtype of lung cancer 1,3. Therefore, developing a novel diagnostic and therapeutic model based on molecular characteristics for LUAD patients is a necessary and feasible approach to promote the individualized precision treatment.

Pyrimidine is the major ingredient of DNA and RNA synthesis, and has been paid attention by oncologists since the advent of chemotherapy, due to its status in interfering with the transmission of genetic information 4. In recent years, as the phenomenon of metabolic reprogramming in the tumor microenvironment (TME) has drawn the great interest of researchers, pyrimidine metabolism has gradually returned to our vision 5,6. In clinical, folate analogues like pemetrexed and nucleoside analogues like gemcitabine, are used to impede tumor progression by disturbing the *de novo* synthesis of pyrimidine nucleotides. Unfortunately, only applying a single chemotherapeutic agent is hard to achieve the desired effectiveness. Hence, it is reasonable to hypothesize that pyrimidine metabolism, not merely *de novo* synthesis, but salvage and degradation pathways, as well as the deposition of pyrimidine metabolites such as thymidine and uridine, may result in potential chemoresistance, thus attenuating the therapeutic benefits of LUAD patients 7. Following this, more efforts are still needed in clarifying the significance of pyrimidine metabolism in the treatment and prognosis of LUAD patients.

In this study, we constructed the pyrimidine metabolism-related signature (PMRS) for LUAD patients by machine learning and deep learning approaches, and investigated its efficacy in prognosis, genomic stability, chemo- and immuno-therapy sensitivity through a comprehensive analysis of multi-omics data, including bulk RNA-sequencing (RNA-seq), single-cell RNA-seq (scRNA-seq), whole-exome sequencing (WES) and metabolomics. Subsequent experiments further validated the most crucial factor in the PMRS model, LYPD3, promoted the malignant progression in LUAD cell lines. Overall, our study provided a novel perspective on pyrimidine metabolism in LUAD patients, and demonstrates that LYPD3 may serve as a potential biomarker and/or therapeutic target for LUAD patients.

## Methods and Materials

### Data collection and processing

We retrospectively collected the expression profiles and corresponding clinical information from 4 independent LUAD cohorts, including 1 RNA-seq dataset from The Cancer Genome Atlas (TCGA, https://portal.gdc.cancer.gov/) and 3 microarray datasets (GSE42127, GSE68465 and GSE72094) from the Gene Expression Omnibus (GEO, https://www.ncbi.nlm.nih.gov/geo/) 8-10. Patients lacking vital status and follow-up time were excluded. Probe IDs were mapped to gene symbols according to annotation files. All microarray data and RNA-seq data were subjected to log_2_ transformation for further analysis. Batch effects among different datasets were corrected by the “Combat” algorithm of R package *sva*. In total, 1,477 patients were included in this study. The detailed clinicopathological characteristics were summarized in **Table S1**. Moreover, the somatic mutation profiles and the copy number variation (CNV) profiles of TCGA-LUAD were accessed from TCGA portal and cBioPortal (https://www.cbioportal.org/), respectively, for genomic characteristics analysis 11.

### Gene set variation analysis (GSVA)

To evaluate the metabolic characteristics of LUAD cohorts, R package *GSVA* was utilized to quantify the metabolic scores based on the gene sets obtained from Kyoto Encyclopedia of Genes and Genomes database (KEGG, https://www.kegg.jp/) 12,13. The corresponding gene sets were provided in **Table S2**. The Harrell's concordance index (C-index) was calculated by R package *survival* and then normalized by min-max normalization 14. The metabolism with the highest average C-index was regarded as the most prognostic pathways in LUAD cohorts.

### Consensus clustering

Consensus clustering performed by R package *ConsensusClusterPlus* was applied for cluster discovery 15. The consensus matrix, the curve of consensus cumulative distribution function (CDF) and the proportion of ambiguous clustering (PAC) statistic were selected to determine the optimal number of clustering. T-distributed stochastic neighbor embedding (tSNE) assay was used for dimension reduction and visualization of the unsupervised clustering results.

### Identification of differentially expressed genes (DEGs) and enrichment analysis

DEGs were identified using the filtering criteria of an absolute value of log_2_ (fold change) > 1 and an adjusted P value < 0.05 by the *limma* package 16. Gene Ontology (GO) and KEGG enrichment analysis of DEGs were performed by the *clusterprofiler* package 17.

### Signature generation and efficacy evaluation

To generate PMRS with excellent accuracy and stability performance, univariate Cox regression was applied to identify prognostic DEGs. These prognostic DEGs were employed to fit prediction models in the TCGA cohort, and then validated across all datasets (TCGA, GSE42127, GSE68465 and GSE72094). The prediction models were comprised of 12 different machine learning and deep learning algorithms, including Ridge/elastic network (Enet)/Lasso regression (*glmnet* package), CoxBoost (*CoxBoost* package), supervised principal components (SuperPC, superpc package), stepwise Cox regression (stepCox, *survival* package), partial least squares regression for Cox models (plsRcox, *plsRcox* package), generalized boosted models (GBM, *gbm* package), random survival forest (RSF, *randomForestSRC* package), survival support vector machine (survival-SVM, *survivalsvm* package), XGBoost (*XGBoost* package) and deep neural network (DNN, *h2o* package) 14,18-26. For each model, the C-indices were calculated across all datasets. The model with the best average C-index was regarded as the optimum.

To evaluate the prognostic performance of the PMRS model, a series of assessment methods were employed, including time-receiver operator characteristic (time-ROC) curve, C-index, integrated area under curve (iAUC) and integrated brier score (iBS). The prognostic efficacy of PMRS in different clinical cohorts was further assessed by the C-index comparison with various clinical factors and previously published LUAD prognostic models.

### Genomic characteristic analysis

Since somatic mutations played a crucial role in genomic characteristics, the mutation spectrum of TCGA-LUAD was analyzed. Among diverse mutational types, Nonsense_Mutation, Nonstop_Mutation, Missense_Mutation, Translation_Start_Site, Frame_Shift_Del, Frame_Shift_Ins, In_Frame_Del, In_Frame_Ins and Splice_Site were considered as non-synonymous mutations, while the remainder were classified as synonymous 27. The landscape and interaction of somatic mutations were visualized by R package *maftools*, and the tumor mutational burden (TMB) was calculated based on the number of mutations per megabase (Mut/Mb) 28,29. According to the best cutoff determined by the *survminer* package, patients were divided into TMB-high or -low groups. The corresponding survival curves were generated by the combination of PMRS and TMB stratification.

Somatic copy number alterations (SCNAs) of TCGA-LUAD were evaluated by GISTIC2.0 in the GenePattern portal (https://cloud.genepattern.org/) 30. Significant amplification (Amp) and deletion (Del) regions along linearized chromosomes between different PMRS subgroups were highlighted based on the CNV frequency, and their locations were determined by the hg38 reference genome file. The SCNAs proportion in PMRS subgroups was calculated based on CNV values generated by GISTIC2.0, in which genes with negative CNV values (-1/-2) were categorized as “Loss” and genes with positive CNV values (+1/+2) were categorized as “Gain”, while genes with a CNV value of 0 were identified as “Neutral”.

Mutations in DNA damage repair (DDR) pathways had been found to impede the repair of deleterious mutations, thereby increasing genomic instability and resulting in the platinum resistance of LUAD patients 31,32. Thus, any non-silent mutations in the DDR pathways, including nucleotide excision repair (NER), base excision repair (BER), homologous recombination repair (HRR), mismatch repair (MMR), Fanconi anemia (FA), non-homologous DNA end joining (NHEJ) and trans-lesion synthesis (TLS) were collected for somatic mutation analysis.

### Drug sensitivity assessment

Drug sensitivity data of GDSC, CTRP and PRISM were obtained from the Cancer Dependency Map Project (DepMap, https://depmap.org/portal/) 33. Both CTRP and PRISM contain AUC values as a measure of drug sensitivity, while GDSC contains the values of half-maximal inhibitory concentration (IC50). Corresponding transcriptome profiles of cell lines were retrieved from the Cancer Cell Line Encyclopedia (CCLE, https://sites.broadinstitute.org/ccle/) 34. The IC50 value for each GDSC compounds was evaluated by R package *oncoPredict* 35. For CTRP and PRISM, compounds with more than 20% missing data were excluded before KNN imputation using the *impute* package, and the AUC value for each compound was predicted by the “calcPhenotype” function. Correlation coefficient between PMRS and estimated IC50 (or AUC) values indicated the potential response of LUAD patients to specific chemotherapeutic drugs.

### Immune infiltration analysis and immunotherapy response evaluation

Tumor immune landscape was depicted by diverse immune infiltration algorithms, including single-sample gene set enrichment analysis (ssGSEA), TIMER, ESTIMATE, CIBERSORT, MCPcounter, xCell and EPIC 36-44. Infiltration levels of immune cells were compared between PMRS subgroups to identify those significantly distinct. Besides, tumor immune-cycle assessed by the Tracking Tumor Immunophenotype (TIP) website (http://biocc.hrbmu.edu.cn/TIP/) and the levels of 15 immune molecules were measured in PMRS subgroups 45,46. Additionally, immune characteristics, such as aneuploidy score, BCR richness, TCR richness, homologous recombination defects (HRD) score, non-silent mutation rate and number of segments were obtained from a pan-immune feature matrix 47.

The potential efficacy of immunotherapy was evaluated using the Tumor Immune Dysfunction and Exclusion (TIDE) score (http://tide.dfci.harvard.edu/) 48. Moreover, two cohorts (GSE91061 and GSE100797), consist of patients who received nivolumab treatment and those who underwent adoptive T cell therapy (ACT), respectively, were utilized to investigate the ability of PMRS in predicting potential immunotherapeutic responses 49,50.

### Single-cell RNA-sequencing analysis

LUAD scRNA-seq dataset GSE131907 was included in this study to elucidate the characteristics of PMRS in the TME. The expression matrices of 11 tumor samples were merged and further processed by the *Seurat* package 51. After removing low-quality cells, expression profiles were normalized by the “NormalizeData” algorithm and 2,000 highly variable genes were identified by the “FindVariableFeatures” function. Principal component analysis (PCA) was applied for dimension reduction based on these genes. Cells were optimally clustered by the “FindNeighbors” and “FindClusters” algorithms. Clusters presented by tSNE reduction were annotated by the *SingleR* package using the “BlueprintEncodeData” reference dataset 52,53. Features of PMRS in the TME were visualized by the “FeaturePlot” function.

Subsequently, epithelial cells were isolated to identify malignant cells. R package *infercnv* was used to estimate CNVs from the scRNA-seq data 54. Macrophages and monocytes were considered non-malignant cells and their CNV patterns were used as a baseline. The cutoff value was set to 0.1 for 10X Genomics. Additionally, the *Monocle* package was used to calculate a pseudo-time trajectory analysis for malignant epithelial cells to infer the differentiation processes 55. The different branches reflected the directions of cellular differentiation.

Additionally, different PMRS patients were identified to investigate their immune characteristics from the viewpoint of single cell analysis. The cellular interactions from various subgroups were evaluated by the *CellChat* package 56.

### Metabolomics analysis

The untargeted metabolomics profiles and expression matrix of LUAD cell lines were obtained from DepMap. Based on the transcriptome profile of the cell lines, they were divided into high-scoring or low-scoring groups for PMRS. Correlations between PMRS scores and metabolite concentrations or KEGG metabolic scores were described by the Mantel test, as implemented in R package* ggcor.* Metabolic distinctions in PMRS subgroups were further illustrated with radar charts.

### Cell culture and transfection

The LUAD cell lines PC-9 and NCI-H1975 were procured from the Shanghai Institute of Biochemistry and Cell Biology (Shanghai, China), and cultured in RPMI-1640 (VivaCell, Shanghai, China) with 10% fetal bovine serum (FBS, VivaCell) at 37℃ in 5% CO_2_. The plasmid *pcDNA3.1-CMV-LYPD3* and an empty vector were purchased from GeneChem (Shanghai, China). The small interfering RNAs (siRNAs) targeting LYPD3 were synthesized by Hanbio Biotechnology (Shanghai, China). Its sequences are listed in **Table S3**. Lipofectamine 3000 (L3000001; Thermo Fisher, MA, USA) was applied for the transfection, according to the manufacturer's protocol.

### Western blot and quantitative real-time polymerase chain reaction (qPCR)

Following the standard protocol, protein samples were extracted with RIPA lysis buffer (P0013; Beyotime Biotechnology) and their concentrations were determined by the bicinchoninic acid (BCA) protein assay kit (P0010; Beyotime Biotechnology). The protein lysates mixed with loading buffer were boiled at 95°C for 5 minutes to destroy their spatial structures. Subsequently, the denatured proteins were separated by 10% sodium dodecyl sulfate polyacrylamide (SDS-PAGE) gel electrophoresis, and further transferred onto polyvinylidene fluoride (PVDF) membranes. PVDF membranes were blocked with 5% skim milk at room temperature for 2 hours, and then incubated with specific primary antibodies overnight at 4℃. After washing with Tris-buffered saline and Tween-20 (TBST) for 3 times, the membranes were incubated with horseradish peroxidase (HRP)-linked antibody for 2 hours at room temperature. Following another three washing with TBST, the immunocomplexes were visualized with the enhanced chemiluminescent kit (BL520A; Biosharp, Anhui, China). Details of antibodies and their corresponding dilution ratios are provided in **Table S4**.

Total cellular RNA was extracted using the RNAeasy™ kit (R0027; Beyotime Biotechnology), following the manufacturer's protocol. Isolated RNA was quantified by NanoDrop2000 (Thermo Fisher) and reverse transcribed into cDNA with HiScript III RT SuperMix (R323-01; Vazyme, Jiangsu, China). The qPCR assay was performed on QuantStudio™ 7 Flex (Thermo Fisher) using ChamQ SYBR qPCR Master Mix (Q331-02; Vazyme). The expression levels of target genes were normalized to beta-actin, and then calculated by the 2^-ΔΔCt^ method. Primer sequences are listed in **Table S5**.

### Cell counting kit-8 (CCK8) assay

For CCK8 assay, transfected PC-9 and NCI-H1975 cells (1×10^3^/well) were seeded into 96-well plates. CCK8 solution (C6005; NCM Biotech, Jiangsu, China) was added to each well and incubated for 1 hour at the appointed time. The absorbance at 450 nm was detected by Multiskan FC microplate photometer (Thermo Fisher).

### Wound healing assay

For wound healing experiment, transfected cells were added to 6-well plates and incubated with serum-free RPMI-1640. When the cell confluence was greater than 90%, the scratch was created with a sterile pipette tip. After washing with PBS, the wound gap at the same location was photographed at the appointed time using a microscope with a 50× magnification (Leica, Hessian, Gremany).

### Transwell assays

For Transwell invasion and migration assays, transfected cells (2×10^4^/well) in serum-free medium were seeded in the upper Transwell chambers (Corning, MA, USA) coated with or without Matrigel (Corning). RPMI-1640 with 10% FBS was added to the lower chamber as a chemoattractant. After incubation at 37℃ for 36~48 hours, cells migrating to the lower layer of the microfiltration membranes were fixed with 4% paraformaldehyde (BL539A; Biosharp) for 15 minutes and stained with crystal violet (C0121; Beyotime Biotechnology) for 30 minutes. The number of cells was then counted under a 200× microscope (Leica).

### Statistical analysis

All statistical analysis and representations were performed using R (version 4.2.3) and GraphPad Prism (version 8.0.1) software. Student's t-test was applied to analyze intergroup differences for variables with normal distribution. Pearson or Spearman correlation analysis was used to assess the correlation between two variables. All *in vitro* experiments were repeated at least three times. A two-tailed P value less than 0.05 was deemed statistically significant.

## Results

### Identification of pyrimidine metabolic subgroups in LUAD

**Figure 1:**
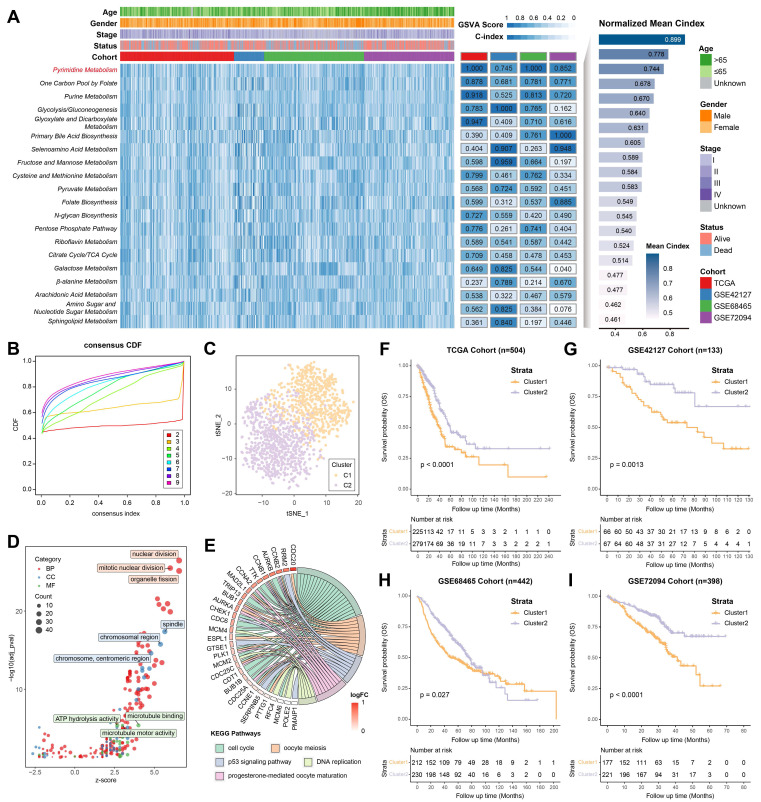
Identification of pyrimidine metabolic subgroups in LUAD. **(A)** The top 20 metabolic pathways with the highest mean C-index across LUAD cohorts. **(B)** The CDF curve for the unsupervised clustering of LUAD patients, k = 2~9. **(C)** The tSNE plot based on the pyrimidine metabolic characteristics. **(D-E)** GO/KEGG analysis among the pyrimidine metabolism-related genes. **(F-I)** Kaplan-Meier survival analysis between the consensus clusters.

According to different metabolic scores, the C-indices of 4 independent LUAD cohorts (TCGA, GSE42127, GSE68465 and GSE72094) was calculated, indicating pyrimidine metabolism had the highest prognostic significance among all metabolic pathways **(Figure 1A, Table S6)**. Consensus clustering was applied for unsupervised clustering and classification of the combined LUAD cohort, where all LUAD samples were divided into k (k = 2~9) clusters **Figure S1A, S1B)**. The CDF curve, PAC score and consensus matrix collectively showed that k = 2 was the optimal number **(Figure 1B, Figure S1C-S1K)**. The tSNE result further revealed significant distinctions in pyrimidine metabolic characteristics between two clusters (C1 and C2) **(Figure 1C)**. GO/KEGG analysis suggested that pyrimidine metabolism-related DEGs were enriched in the cell cycle and DNA replication pathways **(Figure 1D, 1E)**. Furthermore, Kaplan-Meier survival analysis illustrated that Cluster1 showed a superior advantage over Cluster2 across all cohorts **(Figure 1F-1I)**.

### Construction of PMRS

**Figure 2:**
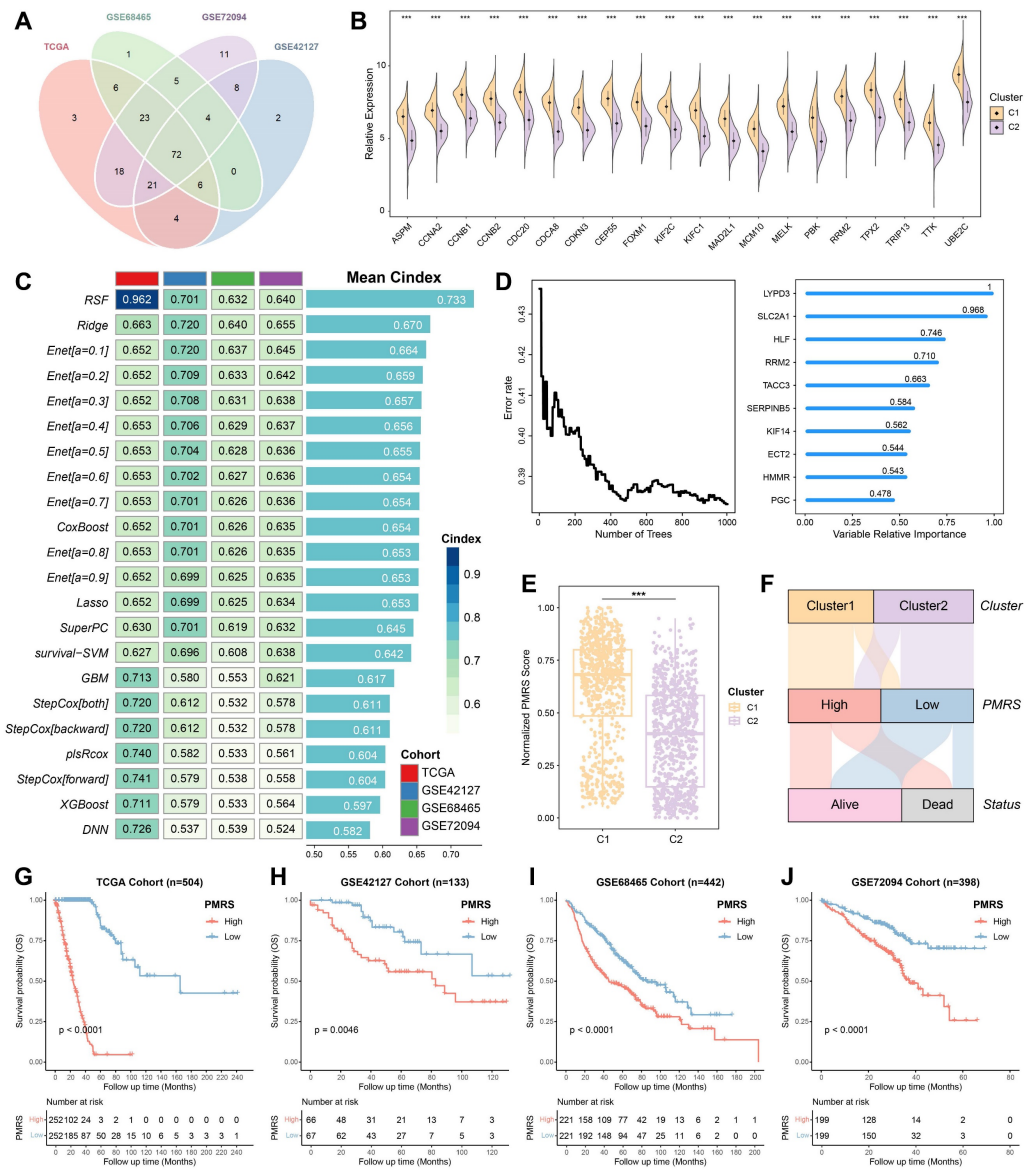
Construction of PMRS. **(A)** The overlapping prognostic DEGs among all cohorts. **(B)** Expression of top 20 prognostic DEGs between consensus clusters. **(C)** An integrated framework of 12 different machine learning and deep learning algorithms was utilized, and then the C-index of each model was calculated across all cohorts. **(D)** The association between error rate and number of trees in the RSF model, and the top 10 most important genes. **(E)** Normalized PMRS scores of consensus clusters. **(F)** The relationship among consensus cluster, PMRS stratification and survival status. **(G-J)** Kaplan-Meier survival analysis between the PMRS subgroups. ***P < 0.001.

The DEGs between pyrimidine metabolic subgroups were subjected to univariate Cox regression analysis, in which 72 prognostic DEGs were identified **(Figure 2A, 2B)**. To investigate the heterogeneity of pyrimidine metabolic subgroups, an unsupervised consensus clustering was performed on these 72 DEGs. Similarly, LUAD patients were also differentiated into two gene clusters **(Figure S2**. Following this, an integrated framework of different machine learning and deep learning algorithms was applied to develop a signature associated with pyrimidine metabolism **(Figure 2C)**. Intriguingly, the RSF model showed the best performance, with the highest C-index (0.733). As the number of trees increased, the error rate of this model gradually decreased. Noteworthy, LYPD3 had the highest importance coefficient among all factors, indicating its role in pyrimidine metabolism and meriting further investigation **(Figure 2D, Table S7)**.

Subsequently, risk scores were calculated based on the importance coefficient of the RSF model. The risk score related to pyrimidine metabolism was termed as PMRS, and all LUAD patients were divided into PMRS-high or -low groups according to the median cutoff. Normalized PMRS scores of different consensus clusters were shown in **Figure 2E**. Sankey diagram further illustrated the relationship among consensus cluster, PMRS stratification and survival status **(Figure 2F)**. In terms of prognosis prediction, patients with higher PMRS tended to have worse prognoses **(Figure 2G-2J)**.

### Evaluation of the PMRS model

**Figure 3:**
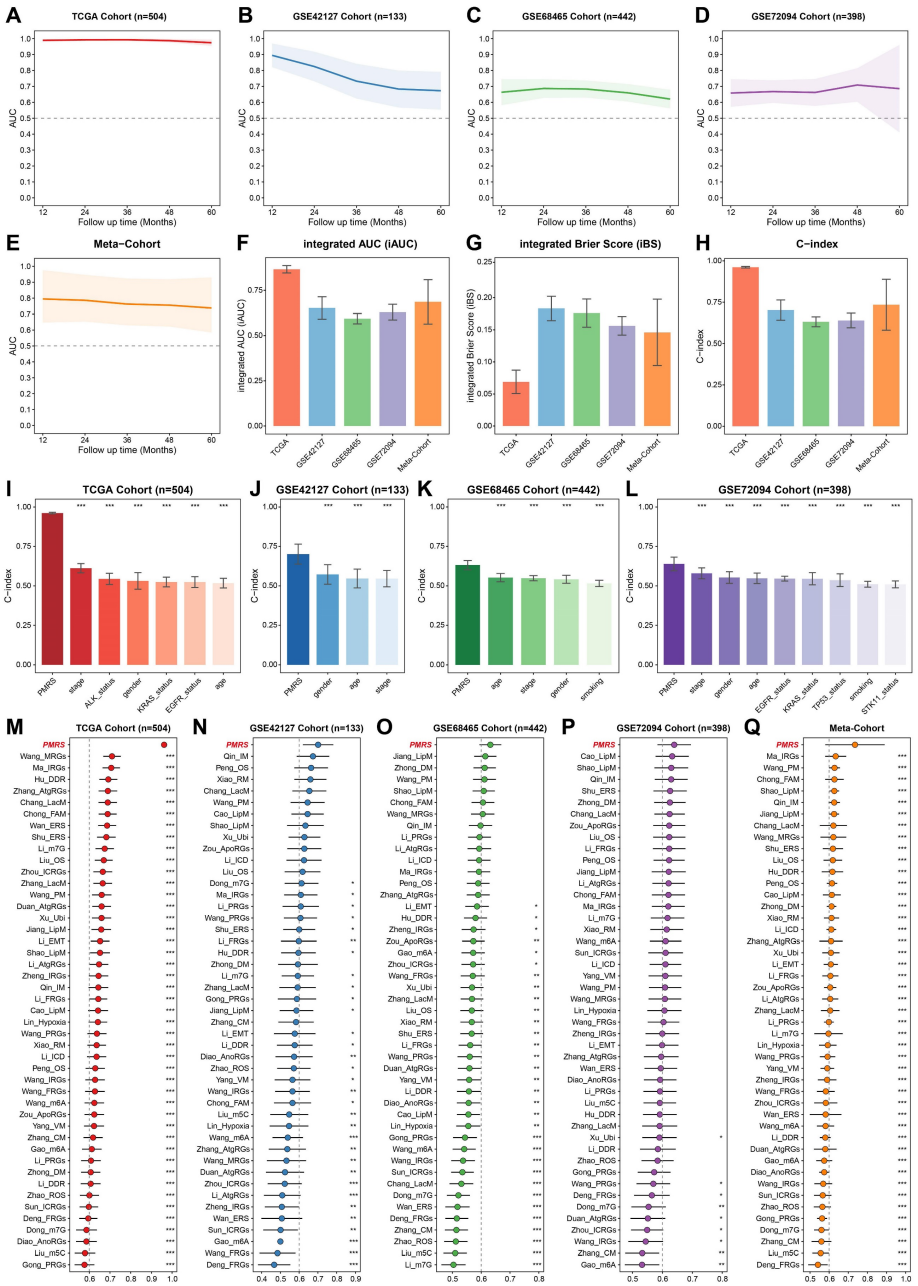
Evaluation of the PMRS model. **(A-E)** Time-dependent ROC curves of PMRS presented with the 1-5-year AUC in TCGA, GSE42127, GSE68465, GSE72094 and Meta-Cohort. **(F-H)** The iAUC, iBS and C-index of PMRS in TCGA, GSE42127, GSE68465, GSE72094 and Meta-Cohort. **(I-L)** The C-index of PMRS and common clinical factors in the TCGA, GSE42127, GSE68465 and GSE72094 cohorts. **(M-Q)** The C-index analysis between PMRS and 45 previously published LUAD prognostic models in TCGA, GSE42127, GSE68465, GSE72094 and Meta-Cohort. Data are presented as mean ± 95% confidence interval [CI]. *P < 0.05; **P < 0.01; ***P < 0.001.

The prognostic predictive capacity of PMRS was initially measured by time-ROC analysis and C-index. Time-ROC curves demonstrated the AUC values of 0.975-0.994 in TCGA-LUAD, 0.673-0.896 in GSE42127, 0.621-0.687 in GSE68465, 0.659-0.709 in GSE72094 and 0.748-0.805 in meta-cohort **(Figure 3A-3E)**. The C-index and 95% confidence interval was 0.962 [0.955-0.968], 0.701 [0.620-0.782], 0.632 [0.594-0.670], 0.640 [0.583-0.696] and 0.735 [0.580-0.891], respectively **(Figure 3F)**. Furthermore, the time-independent indicators, iAUC and iBS, were also employed to evaluate these datasets, demonstrating the excellent predictive accuracy of PMRS **(Figure 3G-3H)**. In addition, we estimated the C-index of common clinical factors, including age, gender, stage and mutation status** (Figures 3I-3L)**. Notably, PMRS exhibited better predictive efficacy than most clinical factors, indicating PMRS was an independent risk factor in all cohorts **Figure S3**. To further validate the prognostic efficacy of PMRS, a total of 45 previously published LUAD prognostic models were collected. These models were constructed based on a variety of biological features, including metabolism, immunity, methylation, ubiquitination, autophagy and novel programmed cell deaths **Table S8**. As expected, PMRS performed better than the majority of the published models across all LUAD cohorts **(Figure 3M-3Q)**. Taken together, PMRS was a valuable prognostic model for LUAD patients.

### PMRS elevates the genomic instability

**Figure 4:**
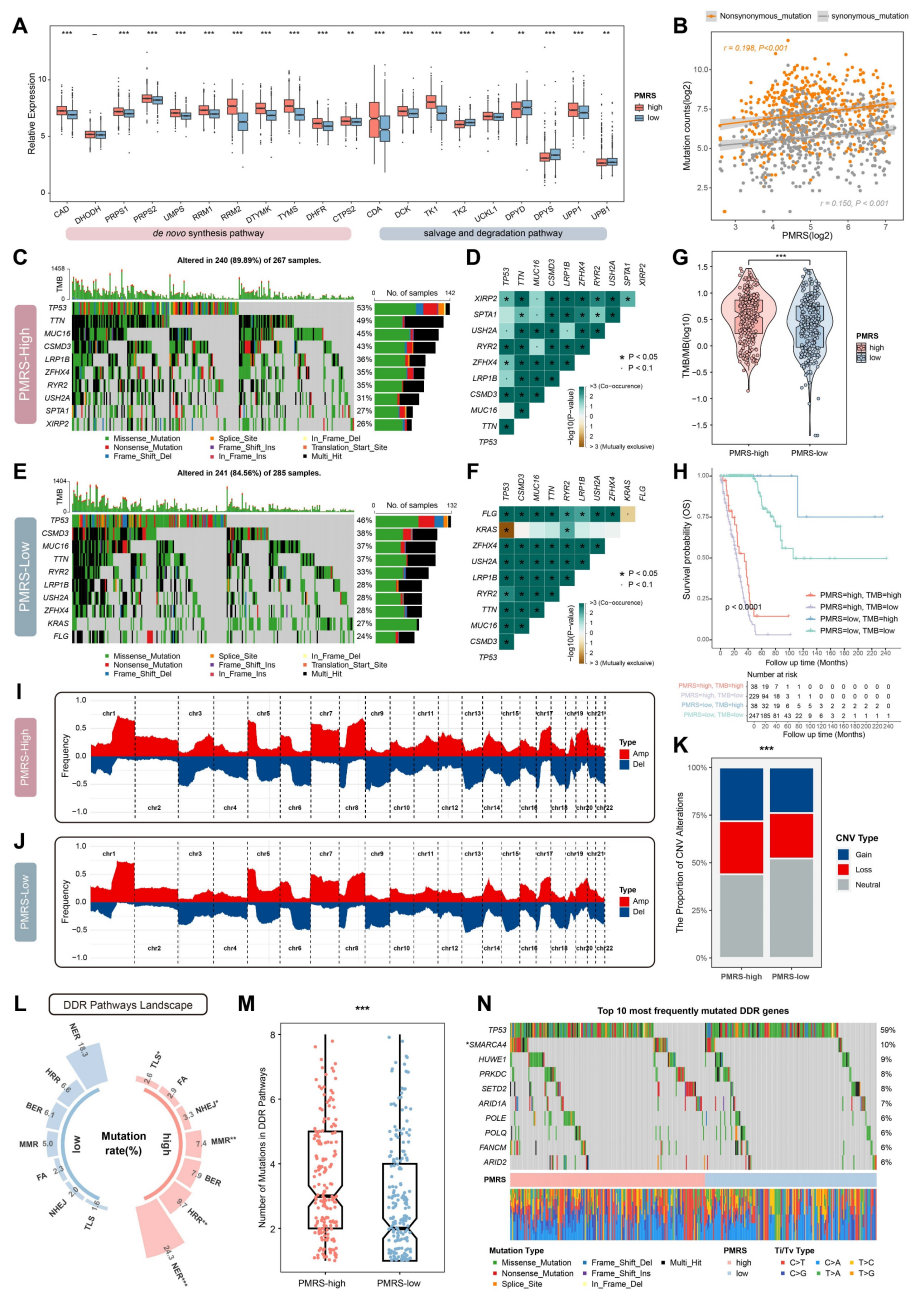
The genomic characteristics analysis between PMRS subgroups. **(A)** The expression profiles of pyrimidine metabolic enzymes involved in *de novo* synthesis, salvage and degradation pathways between PMRS subgroups. **(B)** The correlation between PMRS and non-synonymous and synonymous mutations. **(C-F)** The PMRS-high subgroup showed a higher mutation and co-mutation frequency, according to the maftools analysis. **(G-H)** TMB analysis was conducted for PMRS subgroups, and Kaplan-Meier survival analysis was performed based on the combination of PMRS and TMB stratification. **(I-J)** The CNV profiles of PMRS subgroups were analyzed using GISTIC2.0. **(K)** PMRS-high patients exhibited a higher burden of CNVs in terms of “Gain” or “Loss”. **(L)** Mutation rates of seven DDR pathways (NER, HRR, BER, MMR, FA, NHEJ and TLS) were summarized across different PMRS subgroups.** (M)** Patients with higher PMRS scores had more mutations in the DDR pathways. **(N)** An oncoplot was utilized to display the 10 most frequently DDR mutations in different PMRS subgroups. *P < 0.05; **P < 0.01; ***P < 0.001.

The expression of pyrimidine metabolic enzymes involved in *de novo* synthesis, salvage and degradation pathways were compared between different PMRS subgroups. The results showed that the majority of key enzymes in *de novo* synthesis and salvage pathways were significantly upregulated in the PMRS-high subgroup, whereas the key enzymes related to pyrimidine degradation exhibited the decreased expression **(Figure 4A)**. These findings suggested that patients with higher PMRS scores exhibited more active pyrimidine synthesis in tumors, both in *de novo* synthesis and salvage pathways. Given that pyrimidine was an essential ingredient for DNA synthesis, any abnormalities in the metabolism may affect the DNA replication process adversely, potentially leading to the genomic alterations like somatic mutation and SCNAs 57. Thus, the genomic characterization variants were investigated between PMRS subgroups. The correlation between PMRS scores and non-synonymous (r = 0.198, P < 0.001) and synonymous mutations (r = 0.150, P < 0.001) were revealed in **Figure 4B**. The results of maftools analysis indicated that there was a higher frequency of mutation and co-mutation in patients with higher PMRS scores** (Figure 4C-4F)**. Coincidentally, TMB analysis also performed the similar result **(Figure 4G)**. Survival analysis further illustrated that those patients with higher PMRS scores and lower TMB levels exhibited worse outcomes **(Figure 4H)**. The CNV profiles of PMRS subgroups were analyzed using GISTIC2.0, which revealed that PMRS-high patients had a higher burden of CNVs **(Figure 4I-4K)**. Furthermore, the landscape of DDR pathways between PMRS subgroups was depicted in **Figure 4L**. Patients with higher PMRS scores exhibited more mutations in the DDR pathways **(Figure 4L, 4M)**. An oncoplot was utilized to display the 10 most frequently DDR mutations between different PMRS subgroups **(Figure 4N)**. Among them, TP53, SMARCA4, ARID1A, POLE, ARID2A are involved in the NER pathway, HUWE1, PLOQ in BER, PRKDC in NHEJ, SETD2 in MMR, and FANCM in FA.

### Drug sensitivity assessment for LUAD patients with high PMRS scores

**Figure 5:**
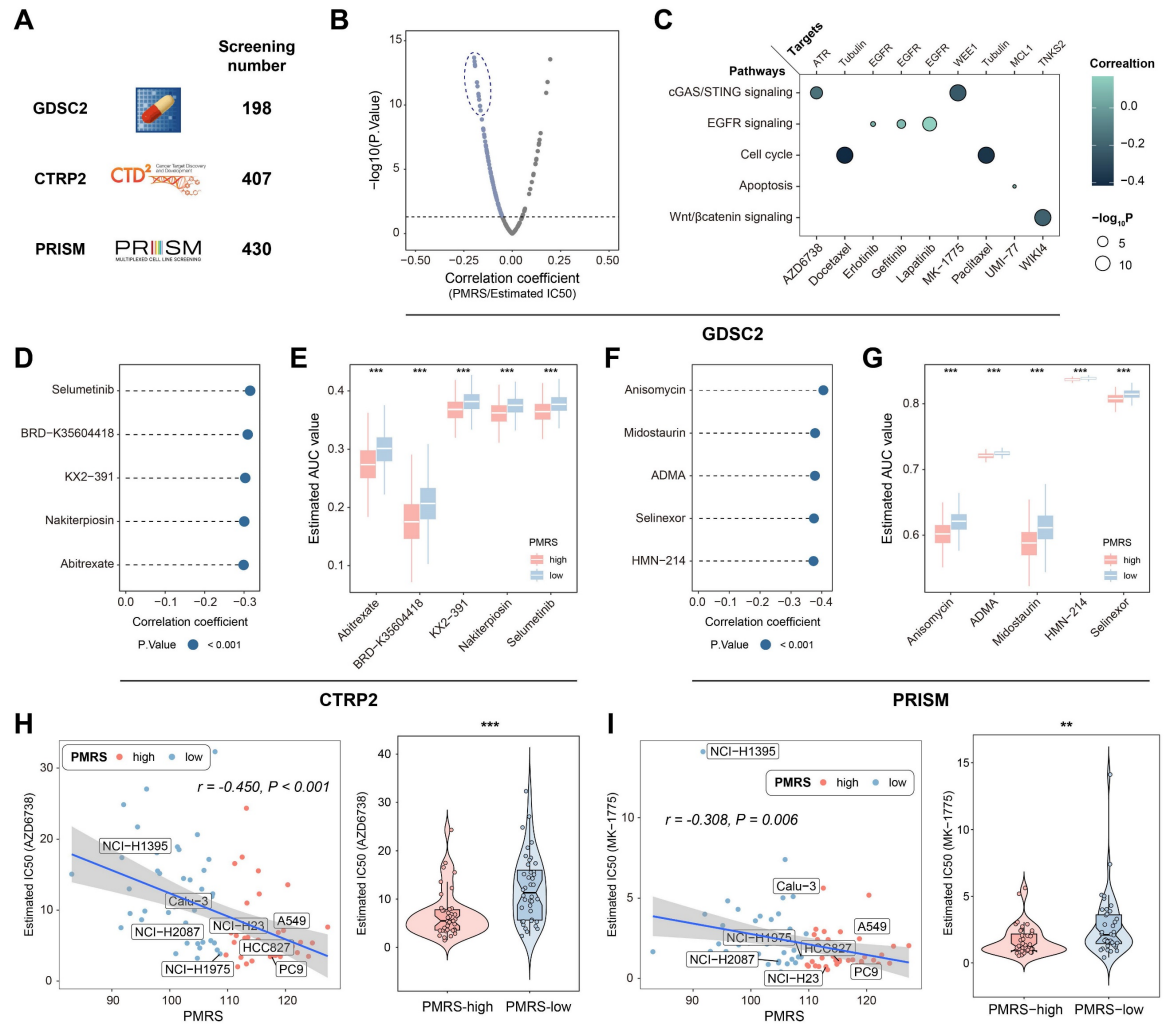
Drug sensitivity assessment for LUAD patients with high PMRS scores. **(A)** A total of 1,035 compounds from 3 drug response databases (GDSC, CTRP and PRISM) were screened to identify potential druggable targets. **(B)** Correlation between PMRS and estimated IC50 values of GDSC drugs. **(C)** The signaling pathways and therapeutic targets of the candidate compounds from GDSC. **(D&F)** Correlation between PMRS and estimated AUC values of 5 drugs with the most negative correlation coefficients from CTRP and PRISM, respectively. **(E&G)** Estimated AUC values of 5 compounds with the most negative correlation coefficients from CTRP and PRISM, respectively. **(H-I)** Correlation between PMRS and estimated IC50 values for AZD6738 and MK-1775 in the LUAD cell lines. **P < 0.01; ***P < 0.001.

Potential druggable targets and related agents might possess therapeutic values for LUAD patients with high PMRS scores. To identify the sensitive drugs for PMRS-high patients, 1,035 compounds from 3 drug response databases (GDSC, CTRP and PRISM) were screened** (Figure 5A)**. Firstly, IC50 values of 198 compounds from GDSC were estimated in all LUAD patients, and correlation analysis was performed on PMRS and IC50 values. The results showed that AZD6738, docetaxel, erlotinib, gefitinib, lapatinib, MK-1775, paclitaxel, UMI-77 and WIKI4 displayed a very strong negative correlation with PMRS scores** (Figure 5B)**. Their signaling pathways and therapeutic targets were presented in **Figure 5C**. For both CTRP and PRISM, 5 drugs with the most negative correlation coefficients and expressions were displayed in lollipop plots and boxplots (CTRP: selumetinib, BRD-K35604418, KX2-391, nakiterpiosin, abitrexate; PRISM: anisomycin, midostaurin, asymmetrical-dimethylarginine (ADMA), selinexor, HMN-214)** (Figure 5D-5G)**. Additionally, the relationship between PMRS scores and the chemosensitivity of above mentioned GDSC drugs was analyzed in LUAD cell lines **(Figure 5H, 5I)**. Intriguingly, AZD6738 and MK-1775 exhibited divergent sensitivity in LUAD cell lines as well. Their correlations with PMRS scores were in accordance with those observed in the clinical cohorts.

### PMRS indicates immunosuppression and immunotherapy resistance

**Figure 6:**
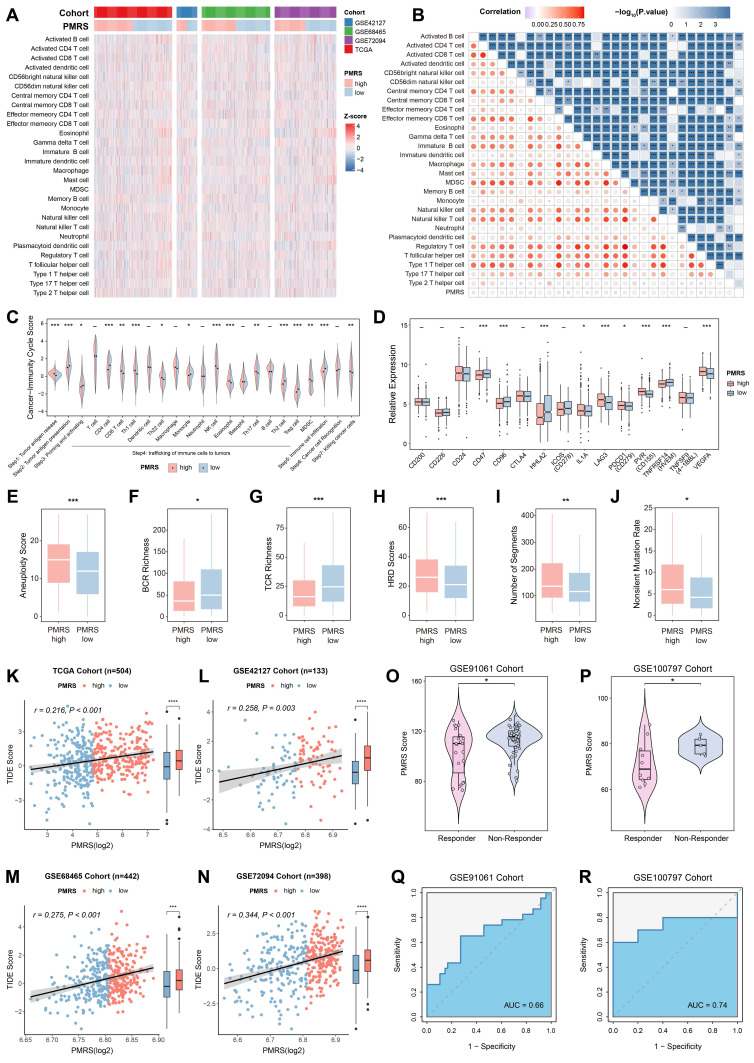
PMRS indicates immunosuppression and immunotherapy resistance. **(A-B)** The relationship between PMRS and immune infiltrations across all cohorts, according to the ssGSEA algorithm. **(C)** Differences in the tumor-immune cycle between PMRS subgroups.** (D)** The expression profiles of immune-related molecules in different PMRS subgroups. **(E-J)** The immune characteristics, including aneuploidy score, HRD score, BCR richness, TCR richness, non-silent mutation rate and segment number were compared between PMRS subgroups. **(K-L)** Correlation between PMRS scores and TIDE scores across all cohorts.** (O-R)** The distribution of PMRS scores between immunotherapy responders and non-responders in GSE91061 and GSE100797 cohorts. *P < 0.05; **P < 0.01; ***P < 0.001.

We depicted the immune landscape of LUAD patients to evaluate the potential role of PMRS in guiding immunotherapy. In most algorithms, significant differences were observed in the infiltration abundance of various immune cells **(Figure 6A-6B, Figure S4)**. Moreover, PMRS-high patients suppressed multiple steps in the tumor-immune cycle, including tumor antigen presentation (Step 2), priming and activation (Step 3), trafficking of immune cells to tumors (Step 4) and immune cell infiltration (Step 5), to maintain immunosuppression **(Figure 6C)**. Meanwhile, immune checkpoints such as PD-L1 and LAG3, as well as immune inhibitory molecules like IL-1A and VEGFA, were overexpressed in patients with higher PMRS scores **(Figure 6D)**. Additionally, several immune characteristics, including aneuploidy score, HRD score, BCR richness, TCR richness, non-silent mutation rate and segment number were compared between the subtypes, revealing the tumor immunosuppressive microenvironment (TIME) of PMRS-high patients **(Figure 6E-6J)**.

Subsequently, the potential of PMRS in predicting immunotherapy responses was assessed by TIDE scores. The results suggested that PMRS-high patients owned higher TIDE scores, indicating they were less sensitivity to immunotherapy and had worse prognoses** (Figure 6K-6N)**. In immunotherapy cohorts (GSE91061 and GSE100797) received diverse interventions, responders had lower PMRS scores than non-responders, and the AUC values for PMRS in predicting immunotherapy responses were 0.66 and 0.74, respectively, indicating a potential correlation between elevated PMRS scores and an adverse response to immunotherapy **(Figure 6O-6R)**.

### PMRS promotes malignant progression of epithelial cells with enhanced pyrimidine metabolism and leads to TIME, from a single-cell perspective

**Figure 7:**
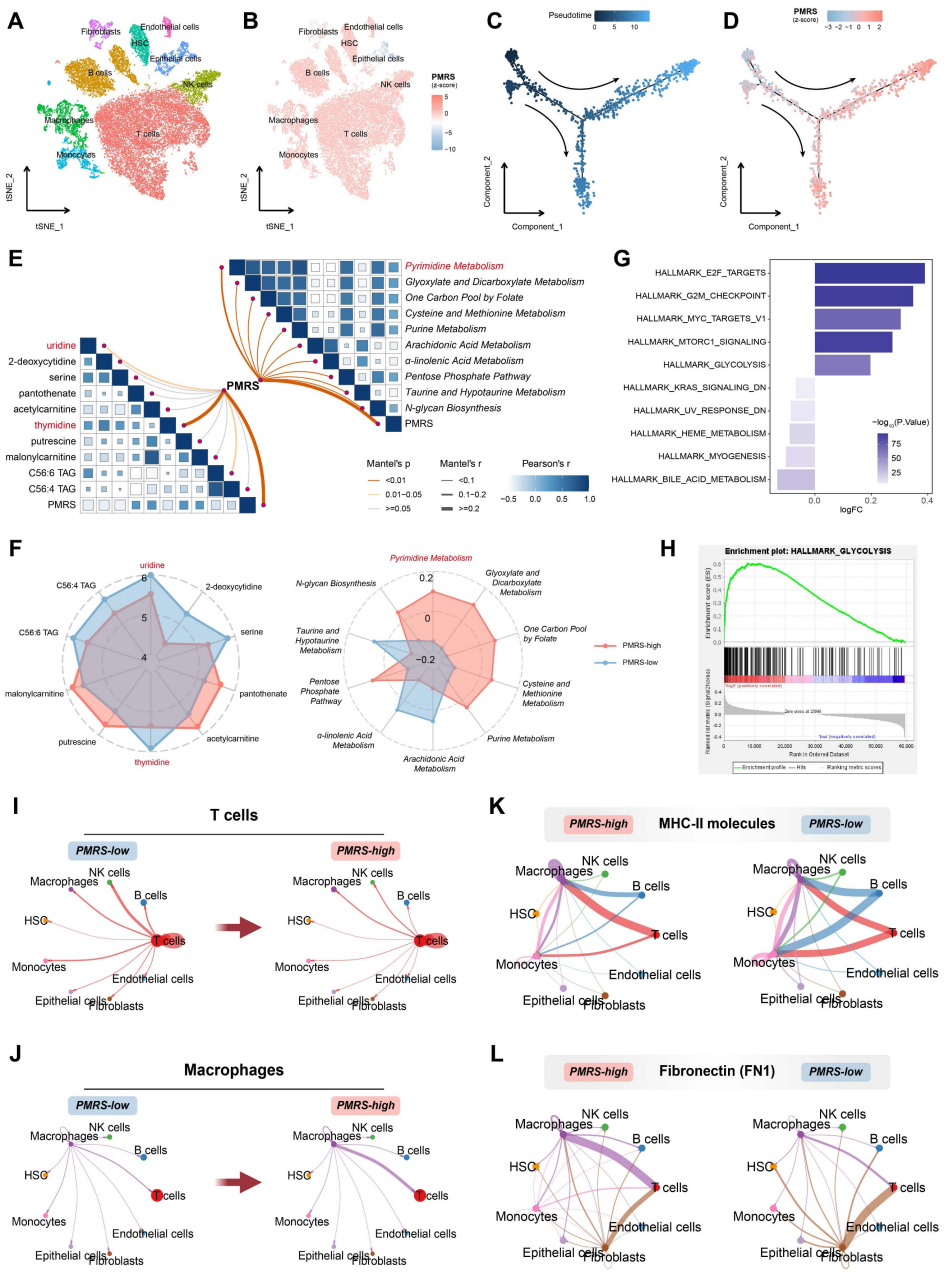
Single-cell analysis of PMRS. **(A)** The tSNE plot displaying the distribution of 9 cell subtypes. **(B)** Visualization of PMRS scores. **(C-D)** Pseudotime trajectory analysis colored by pseudotime and PMRS scores. **(E)** A correlation heatmap illustrated the relationship between PMRS and metabolite concentration or KEGG metabolic scores. **(F)** The radar chart about metabolite concentrations or KEGG metabolic scores between PMRS subgroups. **(G)** Differences in the hallmark gene sets between PMRS subgroups based on GSVA. **(H)** Enrichment of glycolysis in the PMRS-high subgroup. **(I-J)** The cellular interactions of T cells and macrophages with other cells in different PMRS subgroups. **(K-L)** The cellular interactions mediated by MHC-II and FN1 ligand receptors.

LUAD scRNA-seq dataset (GSE131907) was included in the analysis to elucidate the role of PMRS in the TME. 9 distinct cell subtypes were identified, including T cells, B cells, macrophages, monocytes, natural killer (NK) cells, epithelial cells, endothelial cells, fibroblasts and hematopoietic stem cells (HSC) **(Figure 7A, Figure S5A-S5B)**. Their PMRS scores were subsequently calculated across these subtypes. The findings indicated that immune cells such as T and B cells typically had higher PMRS scores, while the PMRS scores of epithelial cells exhibited an obvious gradient distribution **(Figure 7B, Figure S5C)**. Accordingly, the malignant epithelial cells were separated for subsequent pseudo-time trajectory analysis **Figure S5D)**. The results revealed that the malignant epithelial cells with higher PMRS scores appeared later than those with lower scores, suggesting that enhanced pyrimidine metabolism might be an acquired hallmark during the malignant differentiation of LUAD cells **(Figure 7C, 7D)**.

Hence, we performed metabolomics analysis between PMRS subgroups in LUAD cell lines. The results indicated an intimate correlation between PMRS and pyrimidine metabolic scores, as well as thymidine and uridine concentrations** (Figure 7E)**. The lower the PMRS scores patients had, the more uridine and thymidine accumulated in the tumor **(Figure 7F)**. These findings indicated that LUAD patients with high PMRS scores might facilitate the uptake and utilization of thymidine and uridine by activating the salvage synthesis and inhibiting pyrimidine degradation. In addition, the hallmarks of PMRS subgroups were investigated, indicating that multiple carcinogenic pathways were also enriched in the PMRS-high subgroup, such as glycolysis **(Figure 7G, 7H)**.

Furthermore, the immunosuppressive status of PMRS patients was refined from the perspective of single-cell analysis. Cellular interactions of T cells and macrophages with other cells exhibited an attenuation of T cell communication and an enhancement of macrophage communication, as pyrimidine metabolism increased **(Figure 7I, 7J)**. Ligand-receptor comparison analysis revealed the phenomenon of impaired antigen presentation mediated by major histocompatibility complex (MHC) II molecules, as well as increased secretion of inhibitory immune molecules like FN1 in patients with higher PMRS **(Figure 7K, 7L)**.

### Experimental validation of LYPD3 in LUAD cell lines

**Figure 8:**
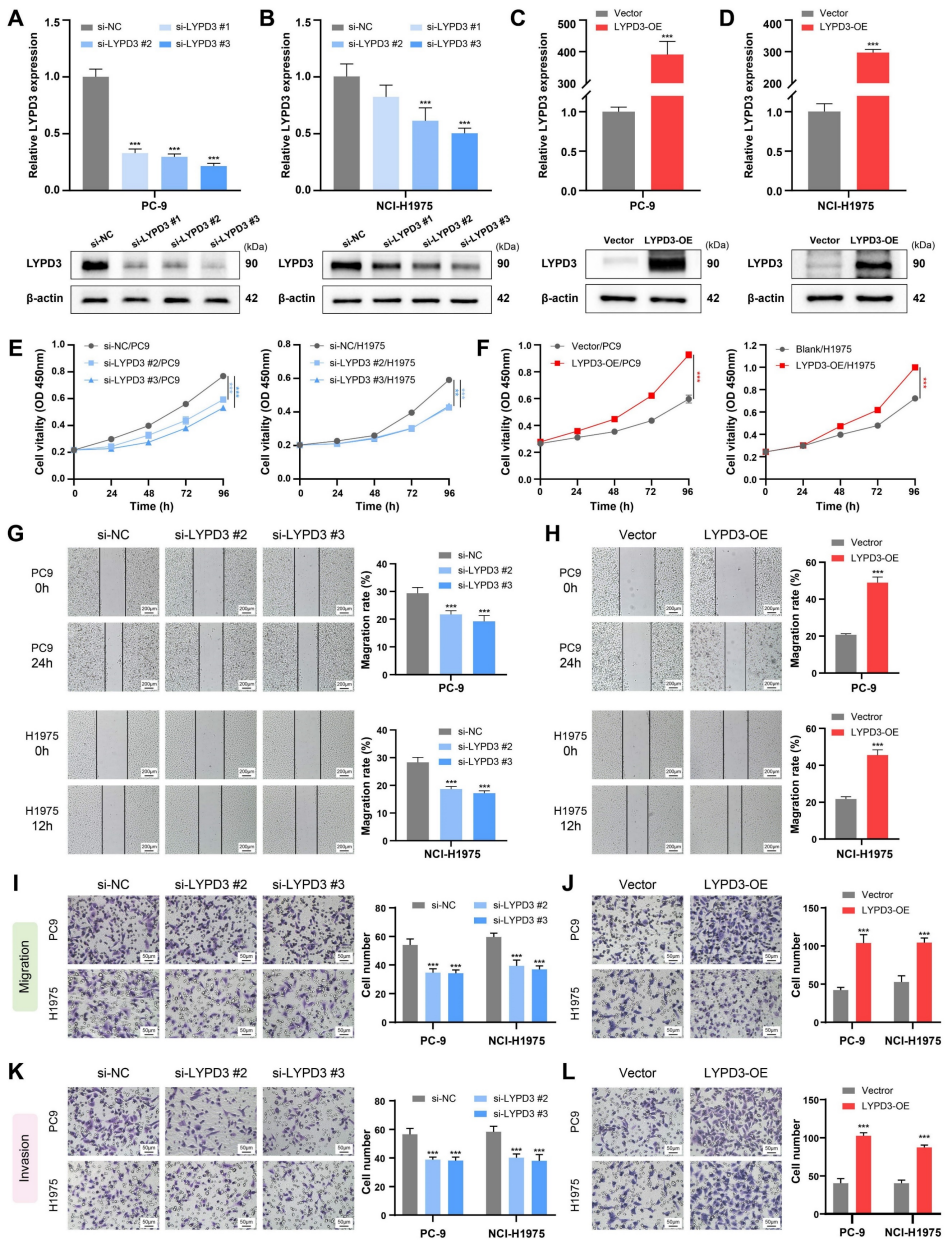
Experimental validation of LYPD3 in LUAD cell lines. **(A-D)** RT-qPCR and Western blot were applied to assess the expression of LYPD3 in PC-9 and NCI-H1975. **(E-F)** CCK8 assay was used to determine the proliferation of PC-9 and NCI-H1975 transfected with si-LYPD3 or *pcDNA3.1-CMV-LYPD3.* **(G-H)** Wound-healing assay was utilized to analyze the migratory activity of PC-9 and NCI-H1975 with the overexpression or knockdown of LYPD3. **(I-J)** Transwell migration assay was used to evaluate the migration of transfected A549 and PC9 cells. **(K-L)** Transwell invasion assay was used to detect invasion after transfection with si-LYPD3 or *pcDNA3.1- CMV-LYPD3*. Data are presented as mean ± 95% confidence interval [CI]. **P < 0.01; ***P < 0.001.

The biological function of LYPD3 was experimentally validated in LUAD cell lines, due to its highest important coefficient in the PMRS model. Two LUAD cell lines PC-9 and NCI-H1975, belonging to PMRS-high and -low subgroups, respectively, were selected for further experiments. RT-qPCR and Western blot demonstrated that siRNA and plasmid successfully regulated LYPD3 expression in PC-9 and NCI-H1975, both at mRNA and protein levels **(Figure 8A-8D)**. si-LYPD3 #2 and #3 were chosen for subsequent functional experiments due to their remarkable knockdown efficiency. The CCK8 assay demonstrated that LYPD3 overexpression promoted the proliferation of LUAD cell lines, while LYPD3 knockdown impeded tumor growth **(Figure 8E, 8F)**. Moreover, the wound healing and Transwell assays elucidated that the overexpression of LYPD3 promoted the migration and invasion of LUAD cell lines, whereas LYPD3 knockdown exerted the opposite effect **(Figure 8G-8L)**. These findings confirmed the pivotal role of LYPD3 in carcinogenesis, as predicted by bioinformatics analysis.

## Discussion

Recently, the phenomenon of tumor metabolic reprogramming has emerged as a prominent area of research. Through altering their metabolic patterns, tumor cells can not only obtain additional energy to sustain survival, but participate in the epigenetic modification or confer immunosuppressive properties to the TME via metabolic by-products (58-60. In this study, we constructed the PMRS model based on pyrimidine metabolism-related genes and evaluated its prognostic value. Our PMRS model worked independently of the existing clinical indicators, and exhibits superior performance in predicting prognosis as assessed by the C-index. Univariate Cox regression also reveals that, apart from PMRS, no other signatures maintain prognostic significance across all cohorts. In addition, we performed corresponding multi-omics analysis, which not only deepened our comprehension of genomic and metabolic landscapes of LUAD patients, but also promoted the development of precision medicine strategies.

Dysregulated pyrimidine metabolism has been reported to result in the potential for DNA damage and mutational bias 57. The PMRS score indicated that there was a notable enhancement in both *de novo* pyrimidine synthesis and salvage pathways, while the activity of pyrimidine degradation was relatively diminished. Moreover, patients with higher PMRS scores exhibited a higher frequency of somatic mutations, SCNAs, as well as a higher levels of TMB. In KRAS/LKB1 co-mutant lung cancer cells, CPS1 depletion led to the DNA damage with an imbalanced purine/pyrimidine ratio 61. Meanwhile, CAD was involved in the clonal evolution in hepatocellular carcinoma, along with the accumulation of SCNAs 62. Subsequently, the mutation landscape of DDR pathways were analyzed 31,63. It indicated that PMRS-high patients had more DDR mutations. Although recent studies suggest that DDR mutations may enhance the immunotherapy efficacy by increasing the neoantigen load, the rapid accumulation of somatic mutations enabled by the aberrant DDR signaling is also non-negligible. This process significantly promotes tumor heterogeneity and clonal diversification 64,65. This might partially explain why PMRS-high patients have higher TMB but have worse survival compared to those PMRS-low patients.

Drug sensitivity analysis of LUAD patients based on PMRS stratification revealed that patients with higher PMRS were more likely to benefit from AZD6738, docetaxel, erlotinib, gefitinib, lapatinib, MK-1775, paclitaxel, UMI-77 and WIKI4. AZD6738 is an ATR kinase inhibitor with a pyrimidine ring structure that targets DDR pathways. Several clinical trials have reported its potential as a monotherapy or in combination with chemotherapy, PARP inhibitors or immunotherapy 66,67. MK-1775, a novel WEE1 kinase inhibitor, were reported to enhance current chemotherapy for its functions during cell cycle and in DNA damage repair 68,69.

Unlike purine metabolism, directly inhibiting peripheral immune cells through its metabolite adenosine, underlying mechanisms of pyrimidine metabolism in the TIME remains poorly understood 70. Our immune infiltration analysis suggested that the enrichment of pyrimidine metabolism increased macrophage infiltration and inhibited the infiltration of T cells, as well as the antigen presentation process. These findings are supported by similar results observed in recent studies. For example, CDA upregulation promoted the accumulation of extracellular uridine diphosphate (UDP), leading to the immunosuppression via P_2_Y_6_ receptors on tumor-associated macrophages (TAMs) 71. While inhibiting DHODH in in *de novo* synthesis, the antigen presentation process was potentiated in a pyrimidine depletion-dependent manner 72. Similarly, the enrichment of pyrimidine metabolism in immune cells also confers therapeutic resistance to cancer cells. Pancreatic ductal adenocarcinoma programmed TAMs not only facilitated their own uptake of gemcitabine by upregulating CDA expression, but also competitively inhibited the uptake of gemcitabine by tumor cells through deoxycytidine secretion 73,74. Hence, it is reasonable to conclude that increased pyrimidine metabolism leads to immune suppression and thus reduces immunotherapeutic efficacy, as predicted by TIDE and immunotherapy cohorts.

Single-cell sequencing and pseudo-time trajectory analysis revealed that PMRS was significantly enriched in the advanced stage of LUAD differentiation, indicating enhanced pyrimidine metabolism might be an acquired hallmark during the malignant differentiation process. Metabolomic analysis of LUAD cell lines indicated an inverse correlation between PMRS and pyrimidine metabolite abundance, implying the salvage pathway was enriched during LUAD progression. Moreover, our findings also revealed the metabolic crosstalk between pyrimidine metabolism and glucose, as well as its clue to immunosuppression.

Lastly, LYPD3 exhibited its significant correlation with malignant phenotypes in both PMRS-high and -low LUAD cell lines. Previous studies have reported that LYPD3 is associated with a poor prognosis in LUAD patients 75. In addition, blocking the downstream pathways activated by LYPD3 and its ligand AGR2 facilitated the progress of endocrine therapy-resistant breast cancer and pancreatic carcinoma 76,77. Other research also mentioned that LYPD3 was involved in the processes of glycolysis and TIME formation in melanoma 78. Our study here revealed the potential link between LYPD3 and pyrimidine metabolism for the first time, suggesting LYPD3 might be a promising biomarker and/or therapeutic target for individualized precision medicine of LUAD patients.

Despite the excellent accuracy and robustness of the PMRS model, this study still has several limitations. First, our study is based on the retrospective clinical cohorts from public databases, lacking the validation from prospective clinical trials. In addition, *in vitro* experiments are limited in the preliminary function of LYPD3. Nevertheless, we are in the process of further validation and investigation. These limitations will be addressed in future research.

## Conclusion

In summary, our study constructed a prognostic signature of pyrimidine metabolic characteristics for LUAD patients, and systematically revealed the role of PMRS in prognosis, genomic stability, as well as chemotherapy and immunotherapy resistance through multi-omics and single-cell analysis. More clinical and experimental studies are expected to validate these findings in the future.

## Supplementary Material

Supplementary figures and tables.
