# Supplementary material for: Multi-omics and single-cell analysis reveals machine learning-based pyrimidine metabolism-related signature in the prognosis of patients with lung adenocarcinoma
Source: Int J Med Sci. 2025 Feb 18;22(6):1375–92. doi: 10.7150/ijms.107694 (PMC11898844; doi:10.7150/ijms.107694)
Supplement: Supplementary file 1 — Supplementary figures and tables. [file ijmsv22p1375s1.zip › Supplementary Figures.docx]

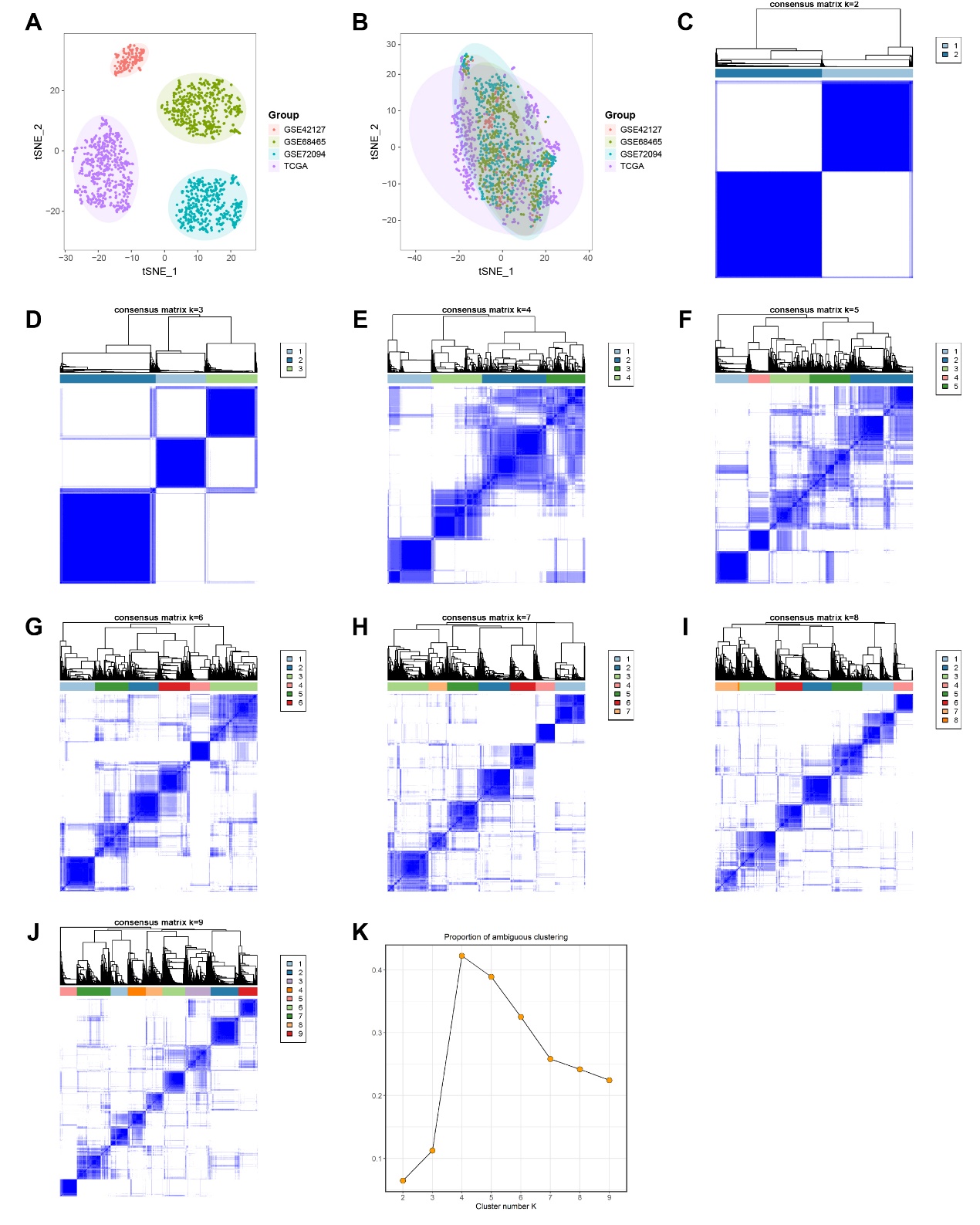


**Figure S1** Consensus clustering of LUAD patients based on the pyrimidine metabolic characteristics. (A-B) The tSNE plots of different cohorts before and after removal of the batch effect. (C-J) Consensus matrixes of LUAD patients for each k (k = 2~9). (K) The PAC score for the unsupervised clustering of LUAD patients, k = 2~9.


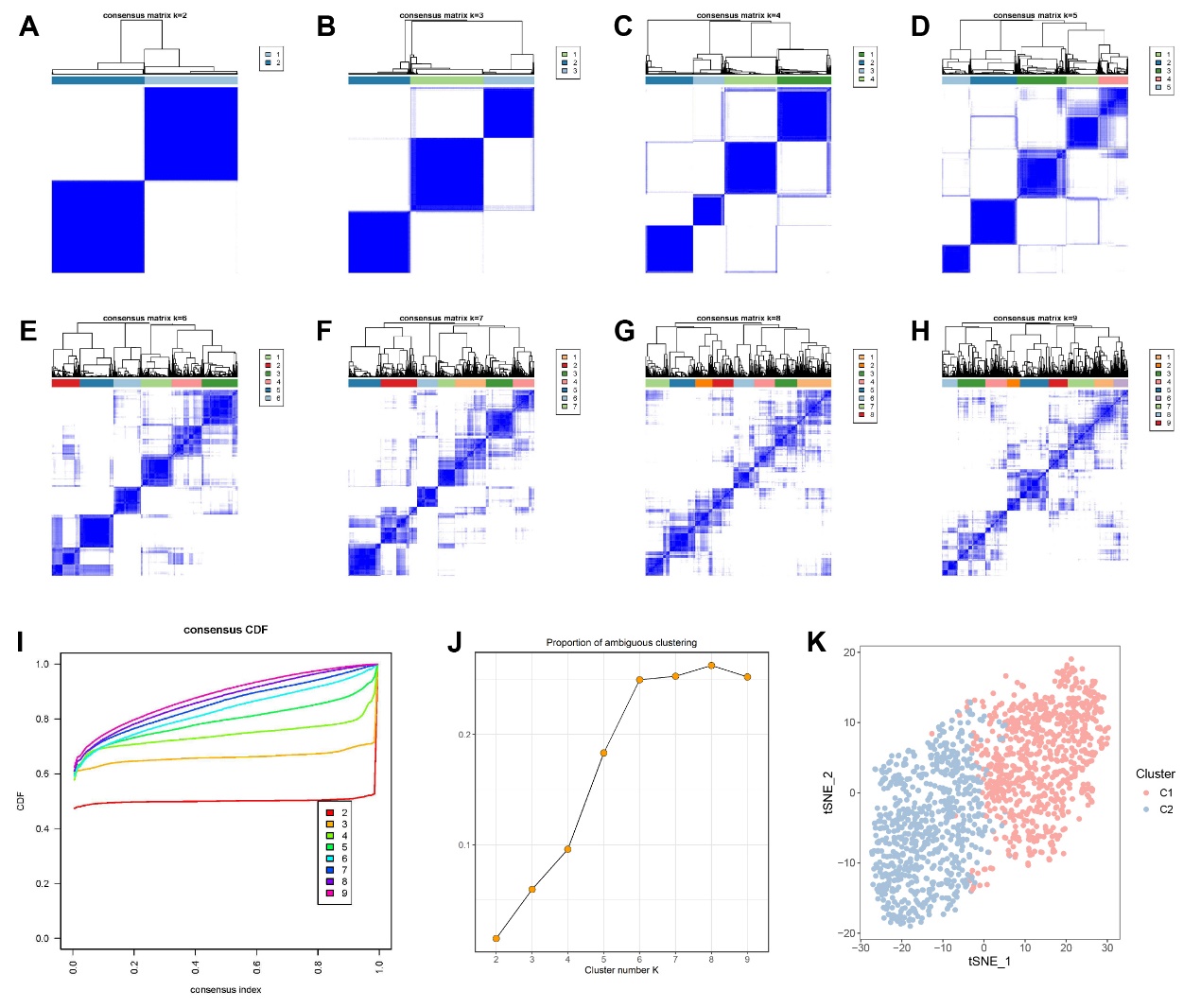


**Figure S2** Consensus clustering of LUAD patients based on the prognostic DEGs. (A-H) Consensus matrixes of LUAD patients for each k (k = 2~9). (I) The CDF curve for the unsupervised clustering of LUAD patients, k = 2~9. (J) The PAC score for the unsupervised clustering of LUAD patients, k = 2~9. (K) The tSNE plot based on the prognostic DEGs.


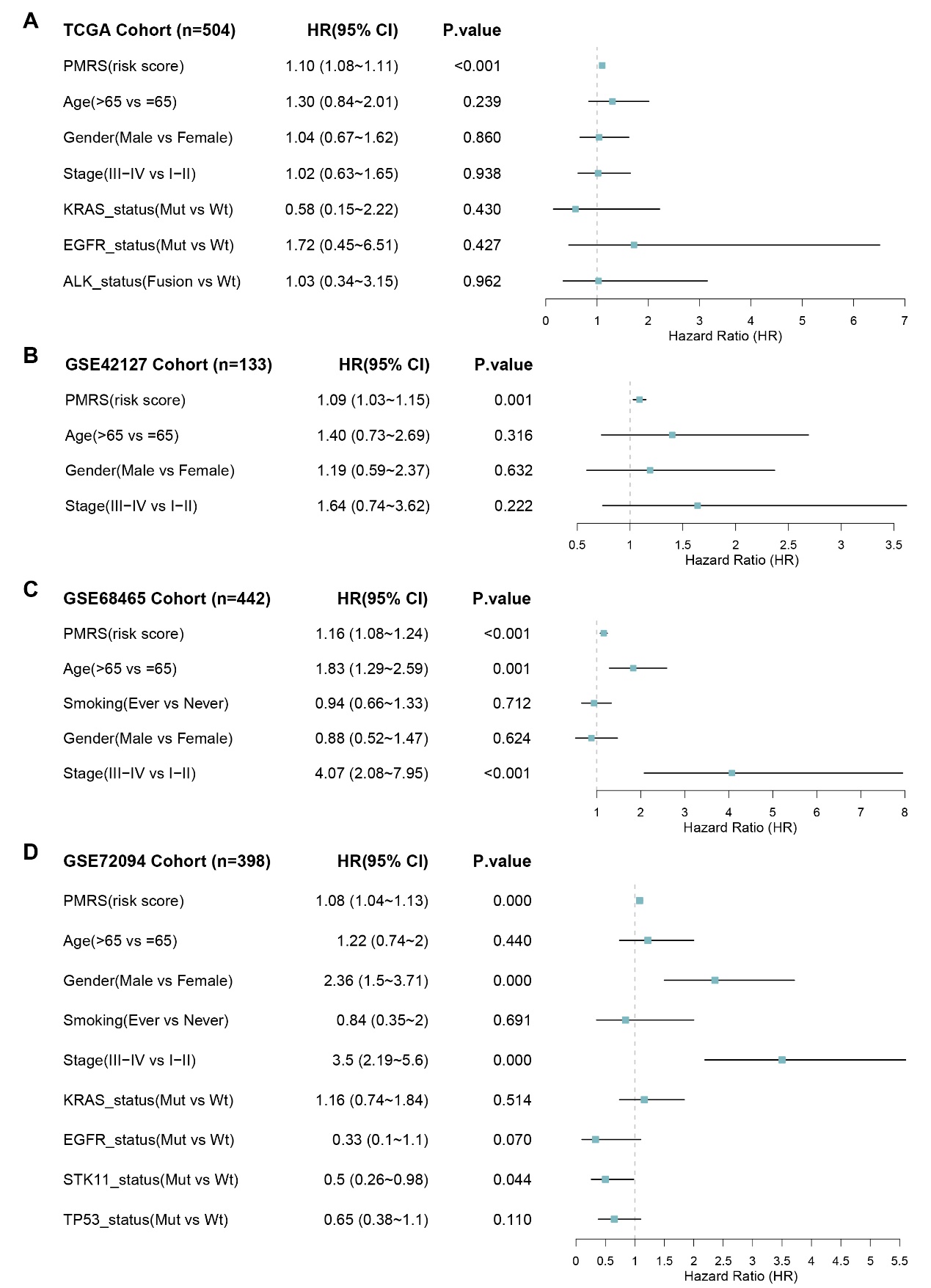


**Figure S3** Multivariate Cox regression of the PMRS model. (A-D) Multivariate Cox regression of PMRS scores and related clinical factors in TCGA, GSE42127, GSE68465 and GSE72094. Data are presented as mean ± 95% confidence interval [CI].


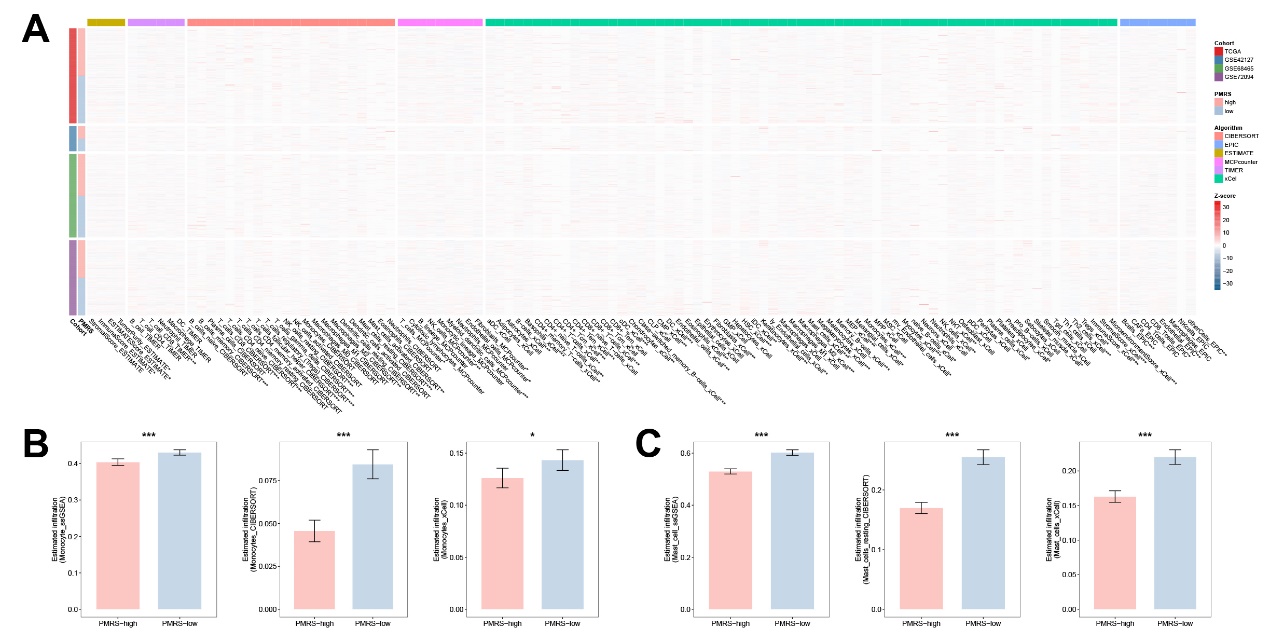


**Figure S4** Analysis of the immune microenvironment between PMRS subgroups in all cohorts. (A) The immune landscape between PMRS subgroups across LUAD cohorts was depicted by diverse immune infiltration algorithms, including ESTIMATE, TIMER, CIBERSORT, MCPcounter, xCell, and EPIC. (B-C) The infiltration of monocytes and mast cells was significantly reduced in the PMRS-high subgroup.


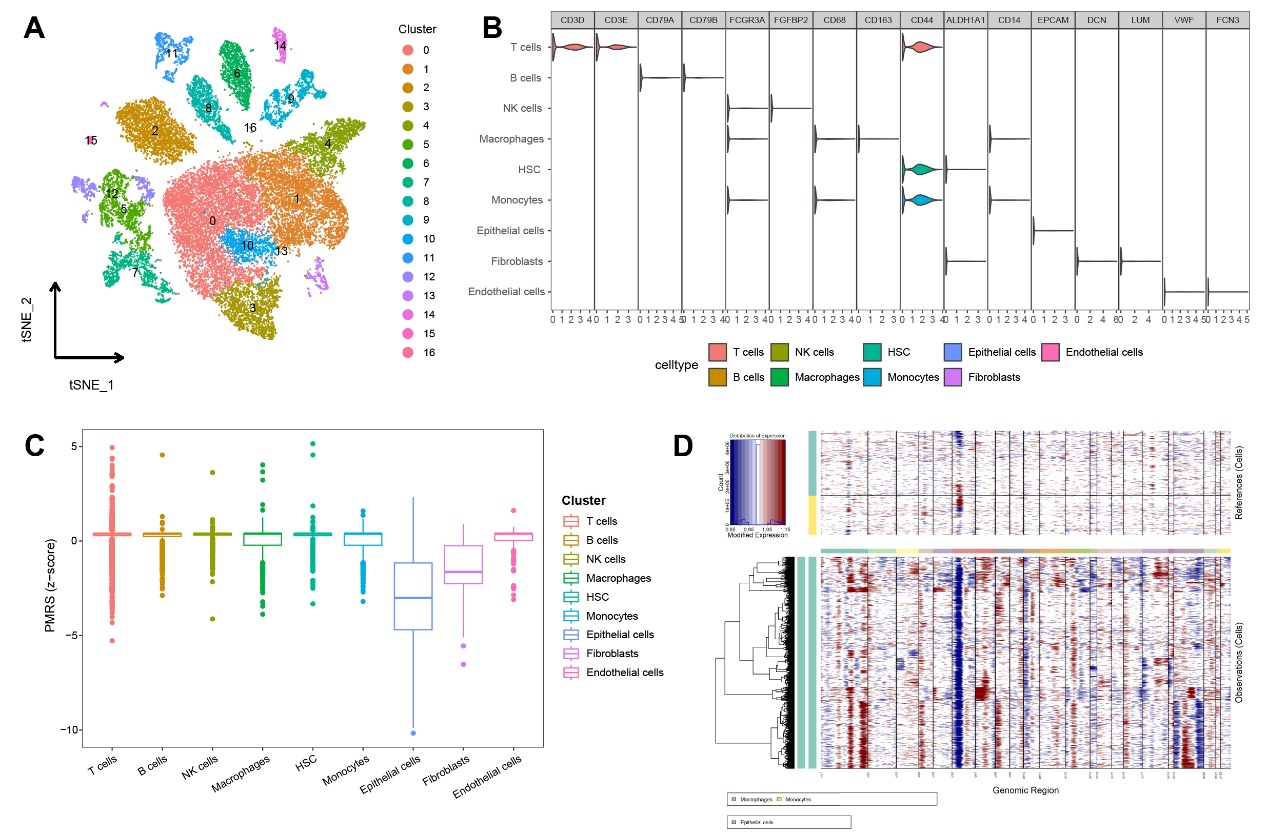


**Figure S5** Exploration of LUAD scRNA-seq data. (A) A tSNE plot colored by 16 different clusters. (B) Marker genes for different cell subtypes. (C) The PMRS scores across all cell subtypes. (D) Malignant cells were divided from epithelial cells by inferring large-scale CNVs with the references of macrophages and monocytes.
